# The Association Between AURKA Gene rs2273535 Polymorphism and Gastric Cancer Risk in a Chinese Population

**DOI:** 10.3389/fphys.2018.01124

**Published:** 2018-08-17

**Authors:** Xiaoyan Zhou, Pengli Wang, Hui Zhao

**Affiliations:** ^1^Department of Oncology, Affiliated Hospital of Shaanxi University of Chinese Medicine, Xianyang, China; ^2^Department of General Surgery, Affiliated Hospital of Shaanxi University of Chinese Medicine, Xianyang, China; ^3^Department of General Surgery, Third Affiliated Hospital of Nantong University, Wuxi, China

**Keywords:** AURKA, gastric cancer, polymorphism, single nucleotide, bioinformatics analysis, Chinese population

## Abstract

The Aurora kinase A (AURKA) gene is frequently amplified and overexpressed in gastric cancer (GC). The overexpression of AURKA promotes inflammation and tumorigenesis in GC. We performed co-expression analysis to identify genes associated with AURKA and speculated its function through the COXPRESdb and STRING databases. We also conducted a hospital-based case-control study involving 385 GC cases and 470 controls in a Chinese population to evaluate the role of AURKA gene rs2273535 polymorphism in the risk of GC. Genotyping was performed using a custom-by-design 48-Plex single nucleotide polymorphism (SNP) Scan™ Kit. Co-expression analysis indicated that the overexpression of AURKA may be associated with poor prognosis of GC. In addition, TT genotypes of rs2273535 polymorphism increased the risk of GC by 72% compared to the AA genotypes. This significant correlation was also observed in the allelic and dominant models. The stratified analysis suggested that TT+AT genotypes showed positive correlation with the risk of GC among female, age <55 years group and non-smokers compared to AA genotypes. In conclusion, AURKA plays an important role in the development of GC. Larger studies with more diverse ethnic populations are needed to confirm these results.

## Introduction

Gastric cancer (GC) remains the second leading cause of cancer death worldwide (Hamashima, 2014). The incidence of GC varies geographically and half of the new cases occur in developing countries (Sitarz et al., 2018). Although the incidence of GC has declined worldwide, prevention of GC should remain a priority. Early detection of both changeable (such as diet, smoking and H. pylori infection) and unchangeable (such as occupational exposures and genetic factors) GC risk factors is vital in primary prevention (Karimi et al., 2014; Daniyal et al., 2015).

The Aurora kinase A (AURKA) gene is frequently amplified and overexpressed in gastrointestinal cancers (Katsha et al., 2013). The overexpression of AURKA promotes activation of NF-kappa B and tumorigenesis (Katsha et al., 2013). AURKA promotes tumor growth and cell survival through regulation of human double minute 22 (HDM)-induced ubiquitination and inhibition of tumor protein 53 (P53) (Sehdev et al., 2014). AURKA promotes signal transducer and activator of transcription 3 (STAT3) activity through regulating the expression and phosphorylation levels of Janus Kinase 2 (JAK2) (Katsha et al., 2014). STAT3 is constitutively activated in human GC, and STAT3 transcriptional activity induces gastric tumorigenesis in the mouse model (Judd et al., 2014).

Aurora kinase A is located in the chromosome 20q13.2 and has 12 exon counts. The rs2273535 polymorphism, located at nucleotide position 91, causes I1e > Phe substitution at amino acid position 31. Two studies have investigated the association between AURKA gene rs2273535 polymorphism and GC risk (Ju et al., 2006; Mesic et al., 2016). However, no study evaluated this association in a Chinese population. Therefore, we conducted this case-control study to explore the distribution of AURKA gene rs2273535 polymorphism in the risk of GC in a Chinese population.

## Patients and Methods

### Subjects

The study consisted of 385 GC patients and 470 healthy controls and underwent surgical resection at the Affiliated Hospital of Shanxi University of Traditional Chinese Medicine (Xian, China) from 2012 to 2016. The diagnoses of GC were all confirmed by histological examination of specimens obtained by endoscopic biopsy or after surgery. GC was histologically grouped according to the Lauren’s classification as either intestinal or diffuse. R classification was used to reflect the extent of GC metastasis. Patients with secondary or recurrent tumors did not meet the inclusion criteria. Totally 470 GC free controls were selected from patients who received regular health examinations at the same period. They were frequency matched (1:1) to the GC cases based on sex and age (±5 years). Individuals with any digestive diseases and tumors were excluded as controls.

The demographic and clinical characteristics of all subjects such as age, gender, Epstein-Barr virus (EBV) infection and smoking status were collected from medical records. The study was approved by the Ethics Committee of the Affiliated Hospital of Shanxi University of Traditional Chinese Medicine and met the standards of Declaration of Helsinki. All subjects have written informed consent prior to the participation.

### Co-expression Analysis

COXPRESdb^1^ is a database that provides co-regulated gene relationships to estimate gene functions (Obayashi et al., 2013). We obtained the co-expressed genes with AURKA through COXPRESdb database, then constructed the protein interaction network of these genes using STRING^2^. Gene Ontology (GO) function and Kyoto Encyclopedia of Genes and Genomes (KEGG) pathway enrichment analyses of these co-expressed genes were carried out in the Database for Annotation, Visualization and Integrated Discovery (DAVID).

### Single Nucleotide Polymorphism Selection

We researched the linkage data from the Ensembl database^3^ and processed it using Haploview software. We also investigated if AURKA gene rs2273535 polymorphism was in strong linkage disequilibrium (LD) with other single nucleotide polymorphisms (SNPs) or if they contributed independently to GC susceptibility, thereby capturing additional significant variants.

### Genotype and Gene Expression Correlation Analysis

Genotype data of AURKA gene rs2273535 polymorphism were available online from the International HapMap Project. Its mRNA expression data were available online from GTEx Portal^4^ (Gong et al., 2018).

### Blood Sampling and Genotyping

Peripheral blood (2 mL) was taken from all patients and controls using test tubes containing ethylene diamine tetraacetic acid. Genomic DNA was extracted from peripheral blood using a QIAamp DNA Blood Mini Kit (Qiagen, Hilden, Germany) according to the manufacturer’s instructions. DNA samples were tested for quality and concentration using an ultraviolet spectrophotometer and stored at -20°C. SNP genotyping was performed using a custom-by-design 48-Plex SNP scan™ Kit (Genesky Biotechnologies Inc., Shanghai, China). This kit was developed according to patented SNP genotyping technology by Genesky Biotechnologies Inc. Recognition of SNP locus allele is achieved with high specificity of the ligase ligation reaction, followed by introduction of non-specific sequences of different lengths at the end of the ligation probe. The ligated ligation reaction was used to obtain the different length ligation products corresponding to the sites. The ligated products were amplified by PCR using universal primers labeled with fluorescence, and the amplified products were separated by fluorescent capillary electrophoresis. Finally, the genotypes of each SNP locus were obtained by GeneMapper software analysis. For quality control, repeated analyses were done for 2% of randomly selected samples with high DNA quality.

### Statistical Analysis

All statistical analyses were performed using Stata 11.0 software (StataCorp, College Station, TX, United States). An analysis of variance and Student’s *t*-test were used to compare the clinical parameters between cases and controls. The proportions of groups were compared by the χ^2^ test. The odds ratio (OR) and 95% confidence interval (CI) were calculated to evaluate the association between the AURKA gene rs2273535 polymorphism and GC risk by logistic analysis. The threshold for significance was set at *P* < 0.05.

## Results

### Bioinformatics Analysis

**FIGURE 1:**
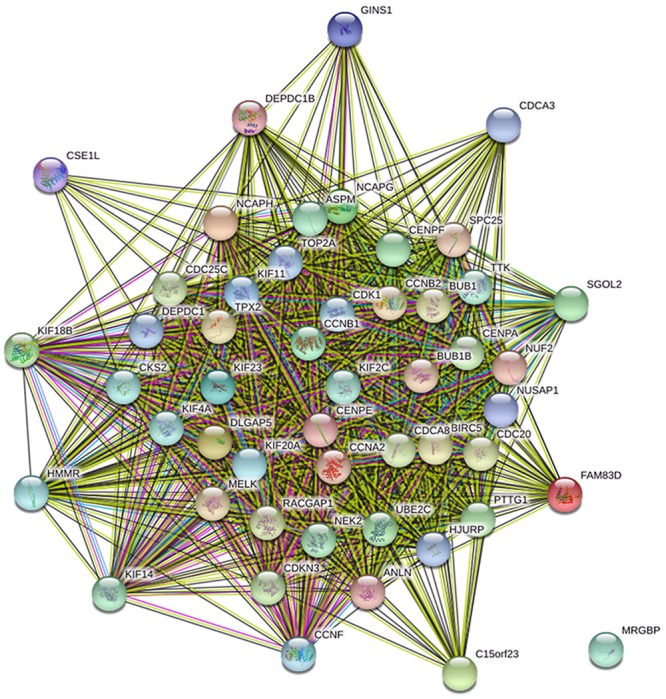
The protein interaction network of genes co-expressed with AURKA. Blue line: from curated databases; Purple line, experimentally determined; Green line, gene neighborhood; Yellow line, text mining.

**FIGURE 2:**
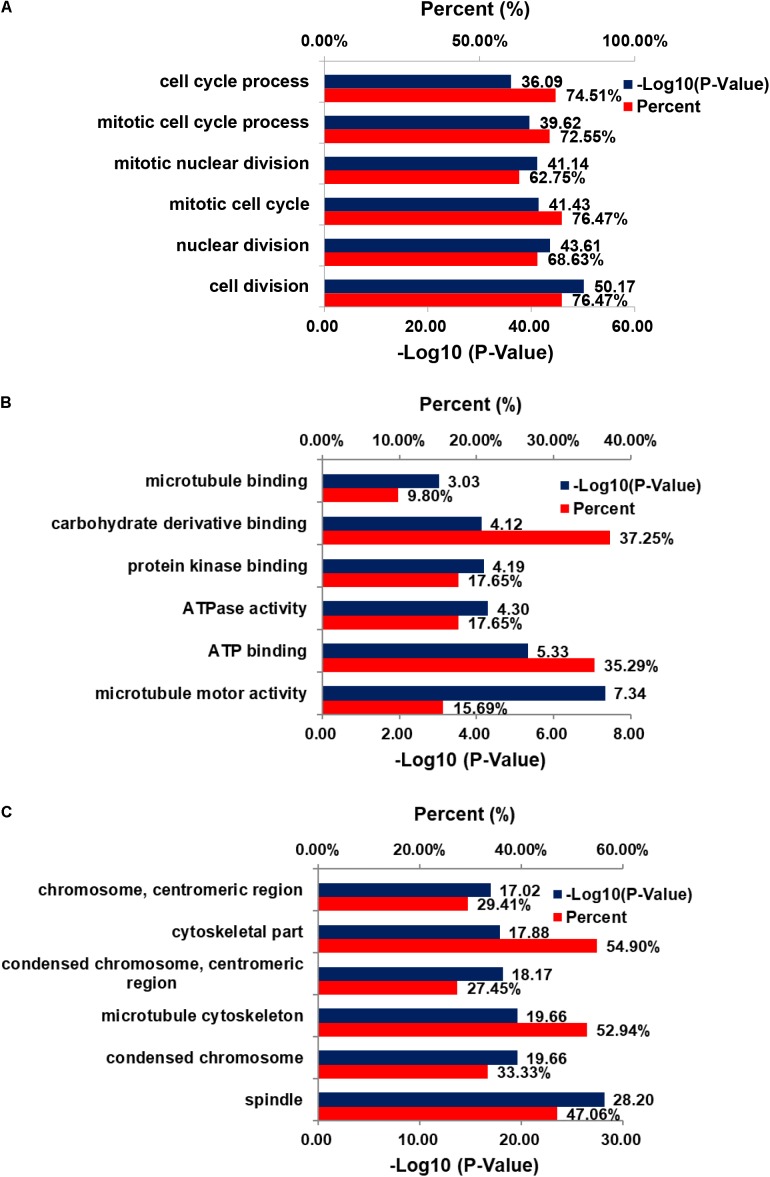
GO enrichment of DEGs. **(A)** Cellular component; **(B)** biological process; **(C)** molecular function.

**FIGURE 3:**
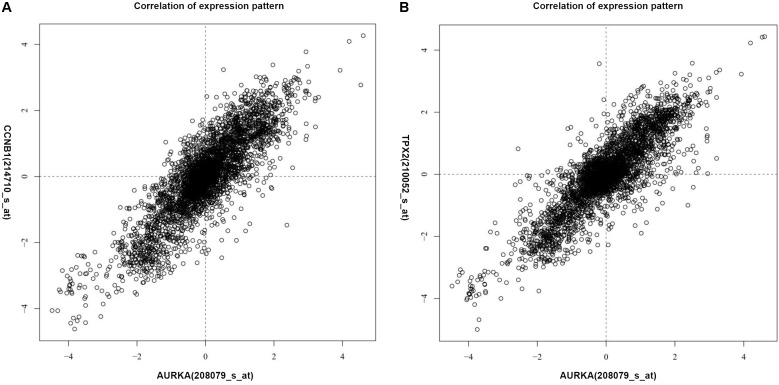
Scatter plots for the mean expression across the AURKA gene and co-expressed gene. **(A)** CCNB1; **(B)** TPX2.

The genes co-expressed with AURKA are shown in **Figure 1**. Go ontology could be divided into three parts [biological process (BP), molecular function (MF), and cellular component (CC)]. They are mainly involved in the term cell division (BP ontology), microtubule motor activity (MF ontology), and spindle (CC ontology) (**Figure 2**). Kegg pathway enrichment analysis indicated that the AURKA co-expressed genes were mainly enriched in pathways like cell cycle (e.g., BUB1, CCNA2, and CCNB1), oocyte meiosis (e.g., AURKA, BUB1, and CCNB1) and progesterone-mediated oocyte maturation (e.g., BUB1, CCNA2, and CCNB12). The five genes most closely related to AURKA were cyclin B1 (CCNB1), centromere protein a (CENPA), discs, large (Drosophila) homolog-associated protein 5 (DLGAP5), TPX2, microtubule-associated (TPX2) and ubiquitin-conjugating enzyme E2C (UBE2C). Scatter plots for the mean expression across the AURKA gene and co-expressed genes are shown in **Figure 3**. There is a strong correlation between AURKA and CCNB1 or TPX2 regarding the mean expression. The overexpression of CCNB1 (Begnami et al., 2010) and TPX2 (Tomii et al., 2017) have been demonstrated to be associated with poor survival in Gc. Further, UBE2C induced epithelial-mesenchymal transition (EMT) by regulating phosphorylation levels of AURKA (Wang et al., 2017). Therefore, we hypothesized that AURKA was related to poor survival of GC. In addition, AURKA and CCNB1 are up-regulated in EBV-transformed lymphoblasts through gene chip (Dai et al., 2012). We hypothesized that AURKA and CCNB1 may be regulated by EBV.

**FIGURE 4:**
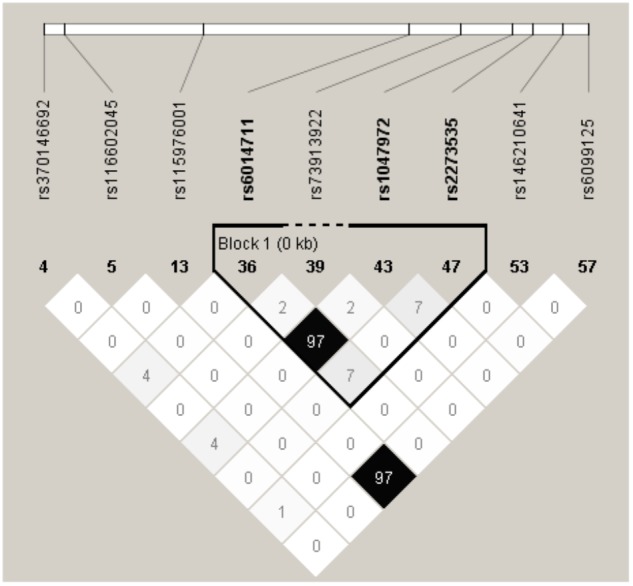
The tagger SNPs in AURKA gene. Linkage disequilibrium (LD) blocks across the locus in Chinese Han. LD value shown: *r*^2^ × 100; *r*^2^ color scheme: *r*^2^ = 0, white; 0 < *r*^2^ < 1, shades of gray; *r*^2^ = 1, black.

Haploview 4.2 software was used to screen tagger SNPs after determining the functional gene. We identified rs2273535 as a tagger SNP, which was not in LD with other polymorphisms. The AURKA mRNA expression levels were regulated by the genotypes of rs2273535 polymorphism (**Figure 4**). We found that there existed a significant difference in the expression levels for rs2273535 polymorphism for the muscle (*P* = 5.9 × 10^−13^).

**FIGURE 5:**
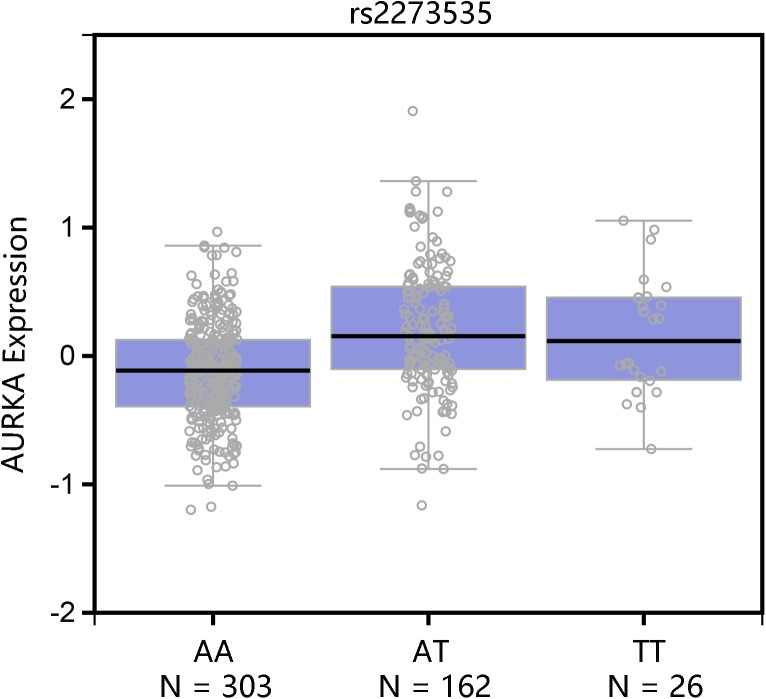
Functional implication of AURKA rs2273535 polymorphism. The genotype of rs2273535 polymorphism and expression of AURKA gene in transformed muscle tissue was based on the public GTEx portal database.

### Characteristics of the Study Population

The demographic and medical data in the case and control groups are shown in **Table 1**. The mean age was 55.12 years and 59.0% of GC patients were male. For the control subjects, the average age was 54.88 and 60.2% were male. There was no significant difference between the two groups concerning gender and age. The percentage of smokers was higher in the patient group than in the control group (*P* < 0.001). Among the 385 cases, 107 (27.8%) were R0; 218 (56.6%) were R1; and 60 (15.6%) were R2. Lauren classification analysis revealed GC intestinal type in 227 cases, diffuse type in 152 cases and 6 were the mixed types. Tubular and poorly differentiated adenocarcinoma accounted for the majority of GC cases. The positive rate of EBV infection in the GC patients was 9.6%, and the control group was 3.4%. The genotype distribution in the controls confirmed to Hardy–Weinberg equilibrium (HWE) (*P* = 0.999), suggesting these subjects could represent the total population.

**Table 1:** Patient demographics and risk factors in gastric cancer.

Variable	Cases (*n* = 385)	Controls (*n* = 470)	*P*
Age (years)	55.12 ± 8.61	54.88 ± 8.70	0.691
Sex			
Female	158 (41.0%)	187 (39.8%)	0.711
Male	227 (59.0%)	283 (60.2%)	
Smoking status			
Non-smoker	124 (32.2%)	231 (49.1%)	<0.001
Smoke	261 (67.8%)	239 (50.9%)	
Epstein-Barr virus (EBV) infection			
EBV-positive	37 (9.6%)	16 (3.4%)	<0.001
EBV-negative	348 (90.4%)	454 (96.6%)	
R classification			
R0 (no cancer infiltration at the margin)	107(27.8%)		
R1 (Microscopic cancer infiltration)	218 (56.6%)		
R2 (Macroscopic cancer infiltration)	60 (15.6%)		
Lauren classification			
Intestinal	227 (59.0%)		
Diffuse	152 (39.5%)		
Mixed	6 (1.6%)		
Histological type			
Papillary adenocarcinoma	23 (6.0%)		
Tubular adenocarcinoma	165 (42.9%)		
Poorly differentiated adenocarcinoma	100 (26.0%)		
Signet ring cell carcinoma	25 (6.5%)		
Mucinous adenocarcinoma	72 (18.7%)		

### Association Between AURKA Gene rs2273535 Polymorphism and GC Risk

The frequencies of the genotypes for rs2273535 polymorphism in cases and controls were shown in **Table 2**. TT genotypes of rs2273535 polymorphism increased the risk of GC by 72% compared with the common genotype AA (TT vs. AA: OR 1.72; 95% CI, 1.02–2.90; *P* = 0.041). Similarly, TT+AT genotypes showed significant correlation with an increased risk of GC compared to AA genotypes. Furthermore, this significant association was also observed in the allelic model (*P* = 0.012). Subgroup analysis revealed that TT+AT genotypes of rs2273535 polymorphism were associated with an increased risk of GC among female, age <55-year participants, non-smokers and EBV-positive patients (**Table 3**). As is shown in **Table 4**, there is no significant difference in genotypes of this polymorphism among different R classifications and Lauren classifications (*P* = 0.167 and 0.190, respectively). However, histological subtypes significantly influence the SNP results (*P* = 0.020).

**Table 2:** Logistic regression analysis of associations between AURKA rs2273535 polymorphism and risk of gastric cancer.

Genotype	Cases^∗^(*n* = 385)		Controls^∗^(*n* = 470)		OR (95% CI)	*P*
	*n*	%	*n*	%		
AT vs. AA	163/182	42.3/47.3	177/261	37.7/55.5	1.32 (0.99,1.76)	0.056
TT vs. AA	36/182	9.3/47.3	30/261	6.4/55.5	**1.72 (1.02,2.90)**	0.041
TT vs. AT+AA	36/345	9.3/89.6	30/438	6.4/93.2	1.30 (0.77,2.21)	0.327
TT+AT vs. AA	199/182	51.6/47.3	207/261	44.1/55.5	**1.38 (1.05,1.81)**	0.020
T vs. A	235/527	30.5/68.4	237/699	25.2/74.4	**1.32 (1.06,1.63)**	0.012

*^∗^The genotyping was successful in 381 cases and 468 controls. Bold values are statistically significant (P < 0.05).*

**Table 3:** Stratified analyses between AURKA rs2273535 polymorphism and the risk of gastric cancer.

Variable	AURKA rs2273535 (case/control)			TT vs. AA	TT vs. AT+AA	TT+AT vs. AA
	AA	AT	TT			
Sex						
Male	120/158	86/103	17/20	1.12 (0.56,2.23),0.748	1.02 (0.50,2.07),0.961	1.10 (0.78,1.57),0.588
Female	62/103	77/74	19/10	3.16 (1.38,7.22),0.007	1.83 (0.80,4.19),0.156	**1.90 (1.24,2.92),**0.004
Age (years)						
<55	80/132	82/81	12/14	1.41 (0.62,3.20),0.407	0.85 (0.37,1.94),0.695	**1.63 (1.10,2.43),**0.016
≥55	102/129	81/96	24/16	1.90 (0.96,3.76),0.067	1.78 (0.88,3.57),0.106	1.19 (0.82,1.72),0.370
Smoking status						
Non-smoker	53/126	56/89	13/16	1.93 (0.87,4.30),0.106	1.29 (0.58,2.89),0.533	**1.56 (1.00,2.43),**0.048
Smoke	129/135	107/88	23/14	1.72 (0.85,3.49),0.133	1.35 (0.66,2.78),0.355	1.33 (0.94,1.90),0.111
EBV infection						
EBV-negative	167/249	155/176	22/28	1.17 (0.65,2.12),0.600	0.89 (0.49,1.62),0.710	1.29 (0.98,1.71),0.073
EBV-positive	15/12	8/1	14/2	**5.60 (1.06,29.59),**0.043	0.88 (0.07,11.24),0.918	**5.87 (1.41,24.39),**0.015

*Bold values are statistically significant (P < 0.05).*

**Table 4:** Comparison of studied data according to AURKA genotypes in all GC cases.

		GC						
		AA (*n* = 182)		AT (*n* = 163)		TT (*n* = 36)		*P*
R classification								0.167
R0	*n*, %	50	27.5%	50	30.7%	7	19.4%	
R1	*n*, %	107	58.8%	83	50.9%	26	72.2%	
R2	*n*, %	25	13.7%	30	18.4%	3	8.3%	
Histological subtype								0.020
Papillary adenocarcinoma	*n*, %	14	7.7%	8	4.9%	1	2.8%	
Tubular adenocarcinoma	*n*, %	72	39.6%	76	46.6%	15	41.7%	
Poorly differentiated adenocarcinoma	*n*, %	51	28.0%	35	21.5%	13	36.1%	
Signet ring cell carcinoma	*n*, %	17	9.3%	5	3.1%	2	5.6%	
Mucinous adenocarcinoma	*n*, %	28	15.4%	39	23.9%	5	13.8%	
Lauren classification								
Intestinal type	*n*, %	79	43.4%	62	38.0%	8	22.2%	0.190
Diffuse type	*n*, %	100	54.9%	99	60.7%	27	75.0%	
Mixed type	*n*, %	3	1.6%	2	1.2%	1	2.8%	

## Discussion

In this study, we hypothesized that AURKA gene expression was associated with poor survival of GC through the comparison of co-expressed genes, although no survival data are available. The genotypes of rs2273535 polymorphism were associated with the expression of AURKA. In addition, AURKA gene rs2273535 polymorphism conferred susceptibility to GC in a Chinese population.

Gastric cancer remains the second leading cause of cancer death worldwide (Hamashima, 2014). The majority of GC is characterized by genetic instability, either microsatellite or chromosomal instability (Hudler, 2012). Expression of AURKA correlates with Wnt-modulator Rac GTPase-activating protein 1 (RACGAP1), which is involved in gastric carcinogenesis (Bornschein et al., 2016). Katsha et al. (2013) found AURKA promotes inflammation and tumorigenesis by activating NF-kappa B signaling pathway. AURKA has also been demonstrated to regulate survivin stability through targeting F-box and leucine-rich repeat protein 7 in GC drug resistance and prognosis (Kamran et al., 2017). We attempted to investigate the association between AURKA expression levels and survival of GC through the comparisons of co-expressed genes. Begnami et al. (2010) found that the expression of CCNB1 was associated with regional lymph node metastasis and poor prognosis. High TPX2 expression was significantly related to poorer disease-specific survival and relapse-free interval (Tomii et al., 2017). It may also hold true for AURKA that AURKA plays an important role in the survival of GC. Annotation results indicated that these co-expressed genes were associated with cell division (BP), microtubule motor activity (MF) and spindle (MF) terms and the pathway of cell cycle. The cell cycle progression is regulated by interactions of specific cyclin-dependent kinases at the G1-S and G2-M checkpoints (Begnami et al., 2010). The cell cycle dysregulation may be involved in the carcinogenesis. Among these co-expressed genes, expression of BUB1 gene correlates with tumor proliferating activity in human GC (Shigeishi et al., 2001). The activation of CCNB1 promotes GC cell proliferation and tumor growth (Shi et al., 2016). We hypothesized that AURKA co-expressed genes may contribute to the risk of GC by regulating cell cycle, which needs further studies to validate it. In according to the GTEx portal database, TT genotypes of rs2273535 polymorphism could cause high expression of AURKA.

Recently, two studies have investigated the association between AURKA gene rs2273535 polymorphism and GC risk (Ju et al., 2006; Mesic et al., 2016). Ju et al. (2006) conducted a population-based study involving 501 cases and 427 controls in the South Korea to evaluate the association between AURKA gene rs2273535 polymorphism and the risk of GC. No association was obtained in the overall analysis (Ju et al., 2006). Subgroup analysis of types of GC also did not observe an association between rs2273535 polymorphism and GC risk among intestinal or diffuse-type GC patients (Ju et al., 2006). Mesic et al. (2016) also performed an overall analysis of this SNP and GC risk with 125 cases and 362 controls in Slovenia. They also did not obtain any significant association in the overall analyses and subgroup analyses by the types of GC and tumor progression (Mesic et al., 2016). To validate the association between this SNP and GC risk in Chinese Han populations, we performed this case-control study. In this study, we found that AURKA gene rs2273535 polymorphism was associated with an increased risk of GC in China with a total of 385 cases and 470 controls. In the subgroup analysis, TT+AT genotypes of rs2273535 polymorphism could increase the risk of GC compared to AA genotypes among female, age <55 year and non-smoker groups. The findings of the above studies were conflicting between this study and abovementioned Caucasian studies. The reasons for these disparities may be as follows. First, there are clinical heterogeneities in different study populations. Differences in genetic background may influence the results. Second, varied geographical environment and eating habits of Asians and Caucasians may also contribute to it. Third, the sample sizes of this study and the Caucasian studies were diverse.

Several limitations of the present study need to be addressed. First, expression data and survival data of AURKA are from the database, and we did not validate its authenticity. Second, the findings of this study were based on unadjusted estimates for confounding factors, which might have affected the final results. Third, we failed to obtain detailed information regarding treatment outcomes, which restricted our analyses. Fourth, this is a hospital-based study and the subjects did not fully represent the general population. Fifth, we could not provide expression data and survival data in this study. Finally, the bioinformatic results were not validated in other studies.

## Conclusion

This study hypothesized that the overexpression of AURKA may be associated with poor survival of GC through the function of genes co-expressed with AURKA. AURKA gene rs2273535 polymorphism could increase the risk of GC. Larger, well-designed studies with ethnically diverse populations are warranted to further validate these findings.

## Author Contributions

HZ conceived and designed the experiments. XZ and PW performed the experiments. XZ and HZ analyzed the data and wrote the paper. PW contributed reagents, materials, and analysis tools.

## Conflict of Interest Statement

The authors declare that the research was conducted in the absence of any commercial or financial relationships that could be construed as a potential conflict of interest.

## Footnotes

1 http://coxpresdb.jp/

2 https://string-db.org/

3 http://www.ensembl.org/index.html

4 https://www.gtexportal.org/home/
